# Effects of Electroacupuncture on *N*-Methyl-*D*-aspartate Receptor-Related Signaling Pathway in the Spinal Cord of Normal Rats

**DOI:** 10.1155/2012/492471

**Published:** 2012-02-08

**Authors:** Ha-Neui Kim, Yu-Ri Kim, Ji-Yeon Jang, Hwa-Kyoung Shin, Byung-Tae Choi

**Affiliations:** Division of Meridian and Structural Medicine, School of Korean Medicine, Pusan National University, Yangsan 626-870, Republic of Korea

## Abstract

This study examined the influence of the N-methyl-D-aspartate receptor (NMDAR) on the modulation of related spinal signaling after electroacupuncture (EA) treatment in normal rats. Bilateral 2 Hz EA stimulations (1-2-3.0 mA) were delivered at acupoints corresponding to Zusanli (ST36) and Sanyinjiao (SP6) in men for 30 min. Thermal sensitization was strongly inhibited by EA, but this analgesia was reduced by preintrathecal injection of the NMDAR antagonist, MK801. Phosphorylation of the NMDAR NR2B subunit, cAMP response element-binding protein (CREB), and especially phosphatidylinositol 3-kinase (PI3K) were significantly induced by EA. However, these marked phosphorylations were not observed in MK801-pretreated rats. EA analgesia was reduced by preintrathecal injection with the calcium chelators Quin2 and TMB8, similar to the results evident using MK801. Phosphorylation of PI3K and CREB induced by EA was also inhibited by TMB8. Calcium influx by NMDAR activation may play an important role in EA analgesia of normal rats through the modulation of the phosphorylation of spinal PI3K and CREB.

## 1. Introduction

Electroacupuncture (EA), a new and modern type of traditional acupuncture, is widely used to treat various types of diseases in a clinical setting with the alterations of peripheral electrical stimulation rather than hand manipulation [1]. EA has an excellent pain relief efficacy profile, and it has been clinically used as a therapy in Korean medicine. Yet, the basis of the pain relief remains unclear.

Basic studies concerning the mechanisms of EA-mediated pain relief have been conducted using an animal model of pain [2]. The induction of EA analgesia involves the N-methyl-D-aspartate receptor (NMDAR), an ionotropic glutamate receptor [3]. The activation of NMDAR plays an important role in the induction and maintenance of hyperalgesia in the spinal dorsal horn [4–6]. Functional NMDAR are heteromeric complexes including the essential NR1 subunit and one or more of the four NR2 subunits (A–D). In particular, the NR2B subunit has an important function in spinal dorsal horn sensory pathways, and phosphorylation of this subunit plays a role in the induction of long-term potentiation (LTP), a phenomenon related to central sensitization [7, 8]. NMDAR containing the NR2B subunit localizes in the extrasynaptic membrane [9]. Their activations are involved in a variety of pain states including the development of central sensitization via the induction of LTP in the dorsal horn of the spinal cord [10, 11].

The induction of LTP requires an increase in the intracellular concentration of calcium in the postsynaptic neuron of the spinal cord [10, 12]. NMDAR-mediated influx of calcium into neurons may initiate the intracellular signaling pathways such as mitogen-activated protein kinase (MAPK) and other related proteins. Thus, NMDAR should be important for signaling cascades in the pain centralization in the spinal cord [13]. Noxious stimulation releases the neurotransmitter glutamate, and the activation of the corresponding glutamate receptors in postsynaptic dorsal horn neurons induces central sensitization [14, 15].

Activation of NMDAR has been implicated in noxious and inflammatory stimulation-evoked extracellular signal-regulated kinase (ERK) and cAMP response element-binding protein (CREB) activation in dorsal horn neurons [15]. The ERK cascades are suggested to contribute to excitatory plasticity in the spinal cord [16]. Activation of intracellular signaling pathways involving p38 and ERK contribute importantly to synaptic plasticity underlying spinal neuronal sensitization. These activations in the spinal cord are reduced by antagonists of NMDAR [17]. The phosphatidylinositol 3-kinase (PI3K) inhibitor inhibits pain-related behavior in a dose-dependent manner and is a major factor in the expression of central sensitization after noxious stimuli [18].

Following concomitant use of EA with NMDAR antagonist, there was a difference in the experimental results between normal and pain animal models. The concomitant use of EA with NMDAR antagonist can synergistically alleviate pain in carrageenan-treated rats [19]. However, following the treatment with NMDAR antagonist, EA analgesia is impaired in normal rats [3]. The present study was performed under the hypothesis that EA analgesia has a different action between normal and pain animal models and produces a basic analgesic effect as a mild nociceptive stimulation.

The aim of the present study was to investigate the role of EA on the NR2B subunit of NMDAR and pain-related signaling proteins in a normal animal model. The link of phosphorylation between the NMDAR NR2B subunit and ERK, p38, PI3K, and CREB was assessed in the spinal cord of normal animal presenting EA analgesia.

## 2. Methods

### 2.1. Animals

Male Sprague-Dawley rats averaging 180 g in weight were obtained from Dooyeol Biotech (Seoul, Korea). The rats were housed at 22°C under alternating 12 hour cycles of dark and light and were fed a commercial diet and allowed tap water ad libitum starting 1 week before the study and continuing throughout the study. All experiments were approved by the Pusan National University Animal Care and Use Committee in accordance with the Council of the International Association for the Study of Pain of December 1982. Each group consisted of six rats for the behavioral test and tree rats for Western and immunohistochemical analysis. All treatments were administered under isoflurane (Choongwae, Seoul, Korea) anesthesia, which was provided using a model VIP 3000 calibrated vaporizer (Midmark, Orchard Park, OH, USA).

### 2.2. EA Stimulation

Under light gaseous anesthesia (1.0% isoflurane in air), two stainless-steel 0.2 mm-diameter needles were inserted to a depth of approximately 3 mm into each hind leg at the acupoints corresponding to Zusanli (ST36) and Sanyinjiao (SP6) in men and were connected to a Pulsemaster Multichannel Stimulator SYS-A300 electrical stimulator (Word Precision Instruments, Berlin, Germany). EA was accomplished with 2 Hz stimulation for 30 min and an intensity set at 1 mA and increasing stepwise to 2 mA and 3 mA, with each step lasting 10 min. For sham-EA control, acupuncture needles were inserted bilaterally at a point lateral to the aforementioned acupoints without any electrical stimulation.

### 2.3. Intrathecal Injection

Intrathecal catheterization was performed as previously described under 1% isoflurane anesthesia [20]. Briefly, a PE-10 intrathecal catheter was inserted through the slit in the L4-5 level of the vertebrate to reach the lumbar enlargement of the spinal cord. Two days after surgery, only those rats without overt signs of spinal cord or root damage, such as paralysis or lameness, were used for experimentation. The NMDAR antagonist MK801 and calcium chelator Quin2 or TMB8 were dissolved in sterile saline and injected intrathecally at a volume of 10 *μ*L via a catheter within 1 min. The catheter was then filled with 8 *μ*L of saline for flushing. Drugs were administered once into the subarachnoid space of the spinal cord 30 min prior to EA stimulation. The vehicle control group for drugs received injections of identical amounts of phosphate-buffered saline (PBS) via an identical method.

### 2.4. Measurements of Thermal Hyperalgesia

The heat paw withdrawal latency (PWL) of six rats each group was measured by the plantar test using a model 37370 apparatus (Ugo-Basile, Comerio, Italy). The rats were placed in six separate cages (17 × 11.5 × 14 cm) for 30 min after EA treatment, and thermal thresholds of the left hind paw were assessed three times with a 5 min interval between trials. The mean values were taken as the PWL. The intensity of the infrared generator was adjusted to produce withdrawal latencies of approximately 8–10 s (80 infrared intensity). A cut-off period of 15 s was used. The latency responses were monitored from 30 min after EA stimulation with or without injection of MK801 and the calcium chelators. Rats not treated with EA were also placed under gaseous anesthesia, and the PWL was then measured.

### 2.5. Western Blot

To examine changes in the NMDAR NR2B subunit, ERK, p38, PI3K, and CREB, the L4-5 segments of the spinal cords were removed 0, 10, 30, 60, 90 and 120 min after the beginning of EA stimulation. L4-5 segment of the spinal cord, 5 mm in length, involved partially lumbar enlargement of the spinal cord, in three rats of each group by laminectomy under anesthesia induced by intraperitoneal injection of 4% chloral hydrate (300 mg/kg). The spinal cords were washed in cold HEPES buffer and homogenized in nine volumes of lysis buffer. Equal amounts of proteins were then separated by 8–12% sodium dodecyl sulfate-polyacrylamide gel electrophoresis (SDS-PAGE), after which the resolved proteins were transferred to a nitrocellulose membrane (Whatman, Dassel, Germany) that was subsequently blocked with 5% nonfat milk in Tris-buffered saline containing 0.4% Tween 20.

The membranes were incubated with anti-NR2B (Millipore, Billerica, MA, USA), anti-phospho-NR2B (pNR2B, ser1303; Upstate Biotechnology, Lake Placid, NY, USA), anti-ERK (Santa Cruz Biotechnology, Santa Cruz, CA, USA), anti-phospho-ERK (pERK, thr202/tyr 204; Cell Signaling Technology, Danvers, MA, USA), anti-p38 (Santa Cruz Biotechnology), anti-phospho-p38 (pp38, thr180/tyr182; Cell Signaling Technology), anti-PI3K (Cell Signaling Technology), anti-phospho-PI3K(pPI3K, Tyr458; Cell Signaling Technology), anti-CREB (Cell Signaling Technology) or antiphospho-CREB (pCREB, ser133; Cell Signaling Technology) for 1-2 h at room temperature, after which the blots were incubated with horseradish peroxidase-conjugated secondary antibody, and the antibody-specific proteins were visualized using an enhanced chemiluminescence detection system according to the recommended procedure (Pierce, Rockford, IL, USA). *β*-actin was used as a loading control for all experiments. Quantification of immunoreactivity corresponding to the total and phosphorylated bands was performed by densitometric analysis using a MultiGauge Version 3.0 (Fujifilm, Tokyo, Japan).

### 2.6. Immunohistochemistry

The L4-5 segments of the spinal cords were removed as described above for Western blot in three different rats in each group. Tissues were fixed in 4% paraformaldehyde and immersed in 30% sucrose for 48 h at 4°C for cryoprotection. Frozen 14 *μ*m-thick sections were then prepared and preincubated in a blocking solution (CAS-block, Invitrogen-Molecular Probes, Camorillo, CA, USA) for 9 min at room temperature. The sections were incubated with the following primary antibodies overnight in PBS at 4°C: pPI3K (Tyr607, ABcam, Cambridge, UK), pCREB (Ser 133, Santa Cruz Biotechnology) and antineuronal nuclei (NeuN, Millipore-Chemicon, Billerica, MA, USA). After being washed with PBS-containing Tween-20 (PBST), the sections were incubated with the secondary antibody, goat anti-rabbit IgG-TR (Santa Cruz Biotechnology), and anti-mouse IgG-FITC (Vector Laboratories, Burlingame, CA) for 2 h at room temperature and then washed with PBST. Slides were mounted in the mounting medium for fluorescence (Vector Laboratories, Inc. Burlingame, CA), and images were captured using a LSM 510 laser scanning confocal microscope (Zeiss, Oberkohen, Germany).

### 2.7. Data Analyses

Data are expressed as the mean ± SEM. Data were analyzed by multifactorial analysis of variance (ANOVA) using the Sigmastat statistical program Version 11.0 (Systat Software, San Jose, CA, USA). Behavioral analysis was performed by a two-way ANOVA post hoc test via Tukey's test. Western blot analysis was performed using a one-way ANOVA post hoc test via Tukey's test. A *P* < 0.05 was considered to be statistically significant.

## 3. Results

### 3.1. Behavioral Analysis on EA with or without MK801 Pretreatment

**Figure 1:**
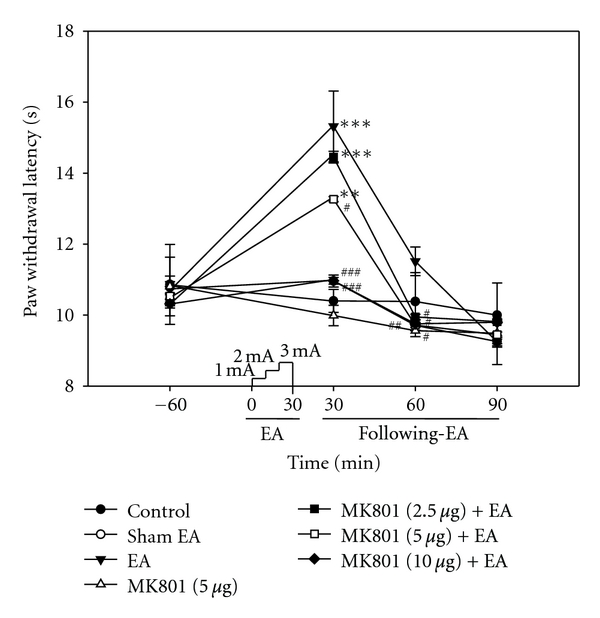
Effect of EA stimulation with or without MK801 pretreatment on the PWL to thermal stimuli. Each point indicates the mean ± SEM (*n* = 6). EA-stimulated rats showed a significant analgesic effect compared with normal control rats, and pretreatment with MK801 inhibited EA-induced analgesia. **P < 0.01 and ***P < 0.001 compared with control rats; ^#^ *P* < 0.05, ^##^ *P* < 0.01, and ^###^ *P* < 0.001 compared with EA-treated rats.

Rats usually resumed full activity within 2–5 min of the cessation of isoflurane anesthesia, regardless of whether they received EA stimulation. Behavioral test measured the basal threshold of heat PWL for the left hindpaw 1 h before EA stimulations, and it was measured at 30 min intervals at 30, 60, and 90 min following EA stimulation. EA produced an analgesia characterized by a markedly higher PWL profile as compared with the normal control rat within 30 min after stimulation. But, findings were similar to control rats 60 min following EA. With EA stimulations following pretreatment with MK801, there was a lower degree of PWL as compared with EA-treated rats; the MK801 effect was dose dependent. Significant differences in PWL were observed in the MK801-treated rats within 30 and 60 min after EA stimulation. These results suggested that the pretreatment with MK801 impaired EA analgesia, which implicated NMDAR in EA analgesia (Figure 1).

### 3.2. NMDAR NR2B Subunit and Related Proteins Analyses on EA with or without MK801 Pretreatment

**Figure 2:**
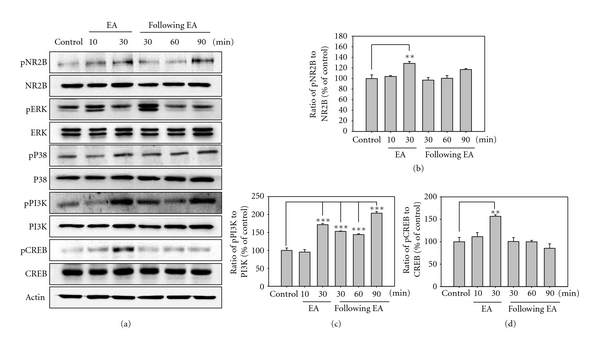
Western blot (a) and densitometric analysis for NMDAR NR2B subunit (b), PI3K (c), and CREB (d) in the L4-5 segment of the spinal cord in EA-treated rats. The level of each protein is expressed as a percentage of the control. Each single experiment was conducted on three pooled animals. The panel represents a typical result from three independent experiments. The phosphorylation of NR2B subunit increased significantly at 30 min from the beginning of EA stimulation. The phosphorylation of PI3K was significantly increased at 30 min from the beginning of EA stimulation and between 30 and 90 min after EA. The phosphorylation of CREB was significantly increased at 30 min from the beginning of EA stimulation. **P < 0.01, ***P < 0.001.

The first experiment examined the induction of the total and phosphorylated NMDAR NR2B subunit, ERK, p38, PI3K, and CREB in normal rats. Next, the NMDAR antagonist MK801 was intrathecally preinjected to identify the proteins associated with NMDAR in the spinal cord and checked for time-dependent alterations. Following EA stimulations, there were no time-dependent alterations in the total protein, but there was a marked degree of changes in the phosphorylated form of NR2B, PI3K, and CREB. The phosphorylation of NMDAR NR2B was significantly increased at 30 min after the beginning of EA stimulation. Phosphorylation of PI3K was significantly increased at 30 min after the beginning of EA stimulation and between 30 and 90 min after EA. Phosphorylation of CREB was significantly increased 30 min after the beginning of EA stimulation. However, phosphorylation of ERK and p38 showed no significant changes (Figure 2).

**Figure 3:**
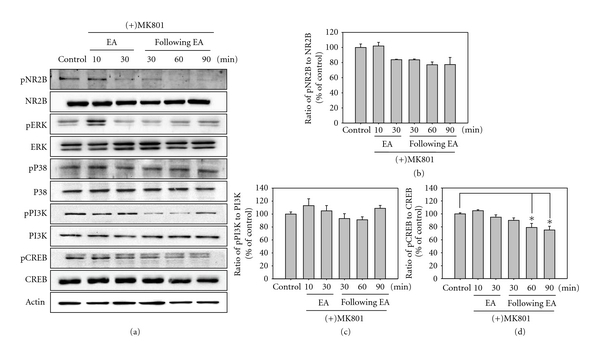
Western blot (a) and densitometric analysis for NMDAR NR2B subunit (b), PI3K (c), and CREB (d) in the L4-5 segment of the spinal cord in MK801- and EA-treated rats. The level of each protein is expressed as a percentage of the control. Each single experiment was conducted on three pooled animals. The panel represents a typical result from three independent experiments. EA-induced phosphorylation of NR2B subunit and PI3K was not observed by MK801 pretreatment, and a significant lower phosphorylation of CREB was detected from at 60 min after EA stimulations; *P < 0.05.

Following treatment with the NMDAR antagonist MK801, the induction of the total and phosphorylated proteins was evaluated. Similar to the above results, there was no marked degree of change in the total protein. The significant alteration of NR2B phosphorylation was not observed at 30 min after the beginning of EA, and this expression was somewhat decreased after EA stimulation. Although there were no significant changes, ERK phosphorylation was increased at 10 min from the beginning of EA. Additionally, the marked increases of PI3K and CREB phosphorylation, which were formed following EA stimulation, were not observed following MK801 pretreatment. Especially, CREB showed a significantly lower phosphorylation from 60 min on following EA stimulations with MK801 pretreatment (Figure 3).

### 3.3. Effects of Calcium Chelator on EA with or without MK801 Pretreatment

**Figure 4:**
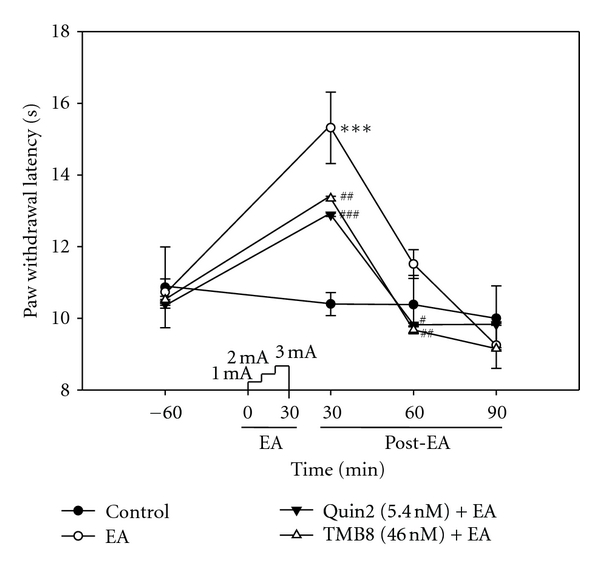
Effect of EA stimulation with calcium chelator pretreatment on the PWL to thermal stimuli. Each point indicates the mean ± SEM (*n* = 6). EA-stimulated rats showed a significant analgesic effect compared with normal arts, but Quin2 and TMB8 pretreatment inhibited EA-induced analgesia. ***P < 0.001 compared with control rats; ^#^ *P* < 0.05, ^##^ *P* < 0.01 and ^###^ *P* < 0.001 compared with EA-treated rats.

**Figure 5:**
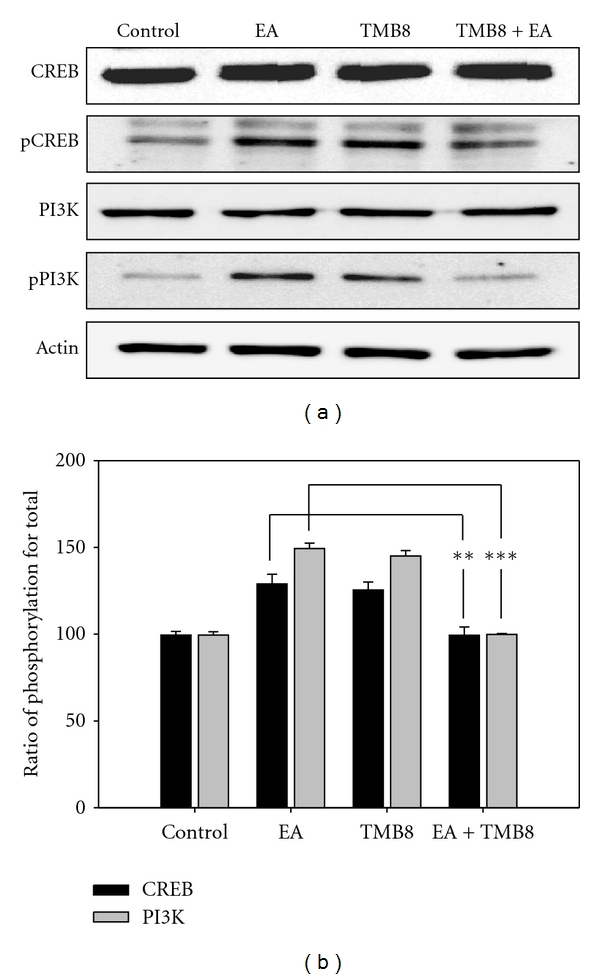
Western blot (a) and densitometric analysis (b) for induction of total and phosphorylation of PI3K and CREB in the L4-5 segment of the spinal cord in TMB8- and EA-treated rats. The level of each protein is expressed as a percentage of the control. Each single experiment was conducted on three pooled animals. The panel represents a typical result from three independent experiments. EA-induced phosphorylation of PI3K and CREB was arrested by TMB8 pretreatment. **P < 0.01, ***P < 0.001.

**Figure 6:**
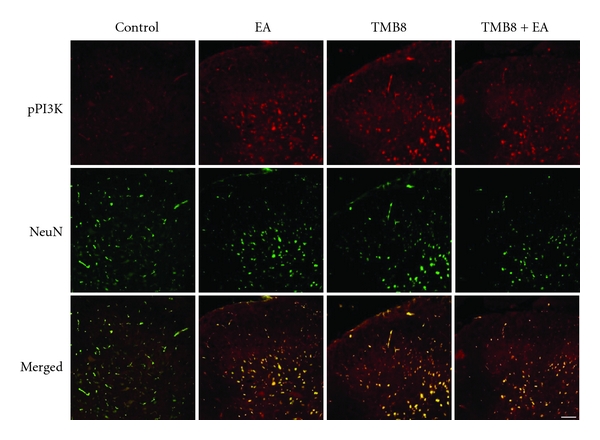
Immunohistochemical localization of pPI3K in the dorsal horn L4-5 segment in normal and EA-treated rats. The expression of pPI3K (red) was usually detected in the laminae IV-VI regions of dorsal horn of the spinal cord. NeuN (green) was also detected in the similar region, and merged image showed that pPI3K and NeuN were colocalized. Scale bar = 200 *μ*m.

NMDAR is a receptor involved in calcium influx into neurons. Accordingly, with the assumption that the above results originated from the intracellular calcium influx via NMDAR, pretreatment with the calcium chelators Quin2 and TMB8 was carried out. Both calcium chelators impaired EA analgesia in a similar manner to the pretreatment with NMDAR antagonist MK801 in the behavioral test (Figure 4). Following the pretreatment with TMB8, changes in the phosphorylation of CREB and PI3K were evaluated at 30 min from the beginning of EA stimulation. Following the pretreatment with calcium chelator, the phosphorylation of CREB and PI3K due to EA stimulations was significantly decreased (Figure 5). To localize pPI3K and pCREB expression and distribution in the spinal cord, we employed immunofluorescence staining with neuron markers NeuN in normal and EA-treated rats. Double-labeling staining showed a large proportion of pPI3K or pCREB and NeuN colocalization in the laminae IV-VI of the dorsal horn (Figure 6). While a similar distribution of pPI3K and pCREB was observed in neuronal cells, strong expression of PI3K was evident in EA-treated rats compared with normal rats.

## 4. Discussion

EA stimulation markedly reduces inflammatory hyperalgesia by inhibiting the release of glutamate in the spinal dorsal horn, and NMDAR antagonists display an antinociceptive action in an inflammatory pain model [19]. Induction of EA analgesia involves NMDAR and is inhibited by a NMDAR antagonist in a normal rat [3]. However, NMDAR-mediated EA-induced analgesic effects, especially the underlying mechanism(s) of EA in normal rats, have received relatively little attention.

To clarify the mechanisms by which EA alleviates pain, studies conducted on normal rats as well as investigations on pain alleviation in a pain model would also be of significance. We performed 2 Hz EA stimulation at ST36 and SP6 acupoints, which previously showed significant analgesic effects and phosphorylation of the NMDAR subunit [21]. The goal of the present study was to observe the time-dependent alteration in the spinal NMDA NR2B subunit, ERK, p38, PI3K and CREB phosphorylation in EA-stimulated rats that had or had not been pretreated with the NMDAR antagonist MK801.

Analgesia induced by EA was observed, and the effects of MK801 pretreatment on heat PWL were assessed. EA stimulation resulted in persistent analgesia within 60 min after EA treatment. However, EA-induced analgesia was significantly abolished by pretreatment with MK801 (Figure 1). Behavioral studies demonstrated that MK801 profoundly inhibited the PWL of EA-induced analgesia, similar to previous observations [3]. Our behavioral results may indicate the involvement of NMDAR in induction or maintenance of EA analgesia.

NMDARs are important in the plasticity of the synaptic processes of the nervous system, such as sensitization of the nociceptive pathways [22]. The inhibition of NMDAR containing the NR2B subunit in the superficial dorsal horn of the spinal cord suppresses nociceptive transmission, and these receptors seem to have a higher conductance than other NMDARs [10, 23]. The NR2B subunit may be important in pain states where a possible build-up of glutamate activates extrasynaptic NMDAR in the spinal cord [9, 10].

NMDAR containing the NR2B subunit plays a role in the development of central sensitization via the induction of LTP in dorsal horn nociceptive synaptic transmission [11]. LTP requires an increase in the intracellular concentration of calcium in the postsynaptic neurons of the spinal cord [12]. NMDARs function as a calcium channel. LTP in dorsal horn neurons are dependent on NMDAR containing the NR2B subunit, and this receptor is involved in use-dependent sensitization at the spinal level [10].

The elevation of intracellular calcium activates a cascade of biochemical events and ultimately leads to altered gene expression [24, 25]. Calcium entry into neurons via NMDAR may initiate MAPKs and the PI3K signaling cascade. Thus, it was appropriate to examine the induction of total and phosphorylated ERK, p38, PI3K, and CREB, as well as the NMDA NR2B subunit during and following EA stimulation.

Phosphorylation of the NMDAR NR2B subunit was significantly induced by EA treatment. In addition, phosphorylation of PI3K, CREB, and especially PI3K was strongly induced by EA stimulation, but that of ERK and p38 was not induced (Figure 2). To demonstrate the possible involvement of NMDAR, the NMDAR antagonist MK-801 was administrated intrathecally before the EA stimulation. EA-induced phosphorylation of the NMDAR NR2B subunit, PI3K, and CREB was strongly inhibited by MK801 pretreatment (Figure 3). These results indicate that EA analgesia may be produced by phosphorylation of PI3K and CREB via NMDAR NR2B subunit activation in the spinal dorsal horn. CREB and PI3K may be important intracellular controllers of EA analgesia in the spinal cord with the NMDAR NR2B subunit.

PI3K is a lipid kinase that generates membrane-associated second messengers, which are able to activate several signaling cascades and cellular processes [18, 26]. PI3K is involved in a transcription-independent and short-term form of spinal plasticity, termed wind-up, which may underlie central sensitization in C-fiber-mediated evoked responses, and PI3K inhibition reduces the phosphorylation of the NR2B subunit of NMDAR [18].

CREB signaling plays a role in the long-term facilitation after noxious stimuli in the spinal cord neurons. CREB phosphorylation represents a better marker than *c-fos *expression for neuronal activity after noxious stimulation because its induction is more rapid and more sensitive [27]. The NMDAR antagonist MK801 markedly suppressed EA-induced CREB phosphorylation in the present study, corroborating the previous demonstrating that MK801-mediated suppression of spinal cord associated pain in a formalin-induced pain model [27].

Influx of calcium via NMDARs leads to the phosphorylation and activation of CaMKII, and CAMKII activation may also affect phosphorylation of the NMDAR NR2B subunit [18]. Therefore, we hypothesized that calcium might be involved in EA analgesia in the spinal cord. To assess this hypothesis, we investigated the effect of calcium chelators on EA analgesia, animal behavior (PWL) and subsequently examined the phosphorylation of PI3K and CREB by Western blotting. A diminished PWL was apparent in rats pretreated with calcium chelator as compared with EA-treated rats; similar results were obtained upon pretreatment with NMDAR antagonist (Figure 4). The phosphorylation of PI3K and CREB due to EA stimulation was decreased by pretreatment with calcium chelator (Figure 5). These results implicate PI3K and CREB as key players in EA analgesia, as in the central sensitization of noxious stimulation. Phosphorylation of the NMDAR NR2B subunit provokes increased calcium influx upon EA stimulation, which may induce PI3K and CREB phosphorylation as sensitization-like mechanisms in the dorsal horn of the spinal cord.

Low-frequency EA activates betaendorphin and enkephalin systems through their receptors, which are expressed in the spinal cord and which contribute to the modulation of nociceptive transmission [1]. The relationship between PI3K activation and EA analgesia in normal rats remains unclear in the nervous system. But PI3K activation contributes to calcium-regulated opioid release from polymorphonuclear cells, the major source of opioids, and thereby inhibits inflammatory pain [28]. Further studies are need concerning the interaction between opioid system and PI3K signaling in spinal nociception during EA stimulation.

Concerning the localization of pPI3K and pCREB induced by EA stimulation, these reactions were colocalized with neuronal marker and were found mainly in laminae IV-VI in the dorsal horn (Figure 6). Primary afferents of low-threshold A*α*/A*β* mechanoreceptors terminate mainly in laminae III–V [29]. These results suggest that EA stimulation induces expression of PI3K and CREB in neuronal cells distributed in laminae IV-VI.

In a pain animal model, calcium influx via NMDAR is involved in the spinal centralization and induces persistent pain. However, depending on the intracellular concentration of calcium, the results might vary. An appropriate level of calcium produces analgesia with the activation of signaling protein. But, an excessively higher level of intracellular calcium contradictorily induces and maintains pain.

In normal rats, the intracellular calcium influx was induced through the activation of NMDAR. Thus, the related proteins were activated, and this led to the EA analgesia. Put another way, as an appropriate mild noxious stimulation, EA stimulation may induce an appropriate degree of intracellular calcium influx by NMDAR and phosphorylate PI3K and CREB, producing EA analgesia.

In the present study, normal rats were sequentially administered EA stimulations at magnitudes of 1, 2, and 3 mA in 10 min intervals and induced EA analgesia over a total period of 30 min. The degree of pain control reached a maximum level when the stimulations were given for approximately 20 min with lower 1 mA in an inflammatory pain model in our lab. In a prior study, only a high frequency of electrical stimulation for C-fibers (3 mA) was capable of activating NMDAR and inducing the intracellular signal pathway in the spinal cord [13]. The frequency and intensity of electrical stimulation may be an important factor to activate the intracellular signal pathway.

 Accordingly, if EA stimulations evoke EA analgesia as a mild noxious stimulation, this would produce an analgesic effect with the application of EA whose magnitude was of an appropriate degree in a dependent manner to the severity of pain. Based on cases not appropriate for a pain model, following the treatment with extremely high degree of stimulations or a long-term treatment with EA, pain might be aggravated.

**Figure 7:**
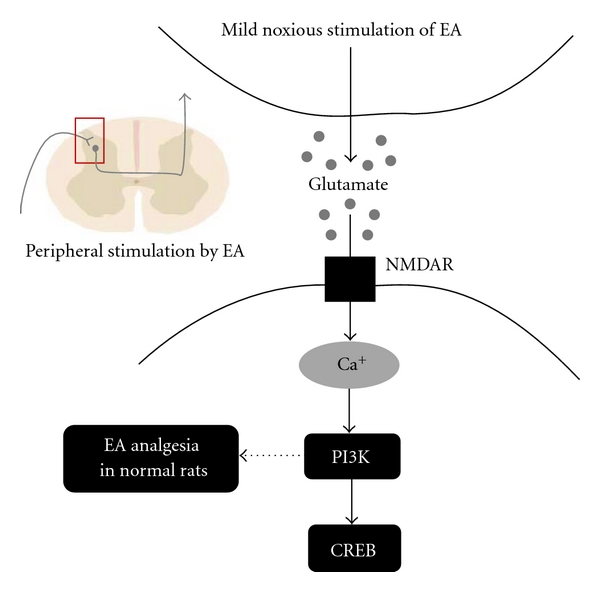
Proposed schematic diagram for of EA-induced analgesia in normal rats. Peripheral stimulation by EA induces glutamate release in the spinal cord dorsal horn this neurotransmitter activates NMDAR. Appropriate influx of calcium via activated NMDAR leads to the phosphorylation of the PI3K and CREB cascade, especially PI3K, manifesting as analgesia. The dotted line denotes where further study is needed to clarify an interaction between PI3K and EA analgesia in normal rats.

Consequently, we suppose that EA analgesia in a normal rat has a different effect on modulating spinal NMDAR-related signaling in rats with inflammatory or neuropathic pain and propose possible schematic diagram (Figure 7). The present results suggest that appropriate influx of calcium via NMDAR in normal rats induces related PI3K and CREB phosphorylation, especially PI3K, manifesting as analgesia. EA analgesia in normal rats may depend on the intensity of the applied EA stimulation. In the application of EA to a pain model, basic studies should also be conducted to examine such parameters as the frequency and intensity of EA depending on the diseases.
